# Family-based association test using normal approximation to gene dropping null distribution

**DOI:** 10.1186/1753-6561-8-S1-S18

**Published:** 2014-06-17

**Authors:** Yuan Jiang, Sarah Emerson, Lu Wang, Lujing Li, Yanming Di

**Affiliations:** 1 Department of Statistics, Oregon State University, Corvallis, OR 97331, USA

## Abstract

We derive the analytical mean and variance of the score test statistic in gene-dropping simulations and approximate the null distribution of the test statistic by a normal distribution. We provide insights into the gene-dropping test by decomposing the test statistic into two components: the first component provides information about linkage, and the second component provides information about fine mapping under the linkage peak. We demonstrate our theoretical findings by applying the gene-dropping test to the simulated data set from Genetic Analysis Workshop 18 and comparing its performance with existing population and family-based association tests.

## Background

When testing genotype-phenotype association using individuals from extended families, one has to account for correlations in genotypes and/or phenotypes between related individuals. One simple and effective method to account for genotype correlations is to simulate the null genotype distribution by gene dropping [1], which is simulating founder alleles according to estimated allele frequencies and dropping these alleles down the pedigrees according to random segregation of gametes (i.e., Mendel's first law). The gene-dropping method is straightforward to implement (e.g., implemented in by Allen-Brady *et al *[2]) and applies to all pedigree structures, but it is computationally intensive and thus is impractical to use when dealing with millions of single-nucleotide polymorphisms (SNPs).

In this article, we derive the analytical mean and variance of the score test statistic under the gene-dropping setting and approximate the gene-dropping null distribution of the test statistic by a normal distribution with the analytically derived mean and variance. Using this normal approximation, the gene-dropping test becomes computationally efficient and can be easily applied to millions of SNPs.

Furthermore, we provide insights into the gene-dropping test by decomposing the test statistic into two components: the first component resembles a quantity frequently used in variance-component based linkage tests and provides information for linkage, and the second component provides information for fine mapping under the linkage peak. Rabinowitz and Laird [3], among others, have pointed out the subtle distinction between two types of null hypotheses in family-based association analysis: the null hypothesis of no linkage and no association versus the null hypothesis of no association in the presence of linkage. To test the latter, one needs to condition on the inheritance $S_{\tau }$ vector at the test locus [3]. Our decomposition provides an explicit separation of linkage and association information in a family-based study.

We compare the performance of the gene-dropping test (using normal approximation) to association tests using only unrelated individuals and to the family-based association test in the software program FBAT [3] by analyzing Genetic Analysis Workshop 18 (GAW18) simulated data set.

## Methods

### Preprocessing of genotype data

We analyzed SNPs from chromosome 3 only. At each of the SNPs, we performed Pearson's chi-squared test for the Hardy-Weinberg equilibrium using 142 unrelated individuals. We excluded SNPs that yielded a *p*-value smaller than 10^−4 ^from our analysis. In the gene-dropping test, we excluded SNPs with estimated minor allele frequency (MAF) smaller than 0.001.

### Preprocessing of phenotype data

We focused on the analysis of the quantitative trait systolic blood pressure (SBP) in the simulated data set 1. The true simulation model was known to us [4]. When testing association between genotype doses and trait values (see later discussion), we include factors AGE, SEX, and AGE by SEX interaction as covariates ($Z_{k}$'s in equation [1]). Including BPMED as a covariate will overcompensate because BPMED is a consequence of SBP level. Instead, we estimated the effect of BPMED from a regression model with only individuals with hypertension. Because BPMED was randomly assigned to individuals with hypertension, the BPMED effect estimated this way will not be biased by its correlation with SBP. We then adjusted the trait values *Y *by subtracting the estimated BPMED effect.

### Score tests of genotype-phenotype association using unrelated individuals

At locus *τ*, we consider a quantitative trait model

(1) $$E\left(Y\right)=\mu +\overset{K}{\underset{k=1}{\sum }}\alpha_{k}Z_{k}+X_{\tau }\beta_{\tau },$$

and test the null hypothesis $\beta_{\tau }=0$. In equation (1), *Y *is the vector of trait values (SBP adjusted for the BPMED effect), *μ *is a constant vector of baseline mean trait values, coefficients $\alpha_{k}$ represent the effects of the covariates $Z_{k},k=1,\ldots ,K,$ (e.g., AGE, SEX and AGE by SEX interaction) on trait values, $X_{\tau }$ is the vector of genotype doses (the number of minor alleles possessed by each individual) at locus *τ*, and the coefficient $\beta_{\tau }$ represents the effect size of a single allele. The fitted value of $\beta_{\tau }$ will reflect the collective effect of all causal SNPs that are in linkage disequilibrium (LD) with the test SNP *τ*[5].

Let *Ŷ *and ${\overset{⌢}{X}}_{\tau }$ be the vectors of fitted values after regressing the *Y *and $X_{\tau }$ on measured covariates $Z_{k}$'s. The score statistic [6,7] for testing genotype-trait association at a single SNP *τ *is $u=X_{\tau }^{\prime }R$, where $R=Y-\overset{⌢}{Y}$ is the vector of residuals. Under the null hypothesis of no association, the variance of *u *is estimated by

(2) $$v=s_{YY}X_{\tau }^{\prime }(X_{\tau }-Z(Z^{\prime }{Z)}^{-1}Z^{\prime }X_{\tau })=s_{YY}X_{\tau }^{\prime }\left(X_{\tau }-{\hat{X}}_{\tau }\right),$$

where $Z=\left(1,Z_{1},\ldots ,Z_{K}\right)$ and $s_{YY}$ is the sample variance of the residual trait values (1 is a vector of ones) [6]. To test association, $u^{2}/v$ is compared with a $\chi_{1}^{2}$ distribution.

### Family-based association test by gene dropping

When related individuals are used to compute the score test statistic $u=X_{\tau }^{\prime }R$, components of $X_{\tau }$ can be dependent, and the variance estimator (2) is no longer valid. One can account for correlations between components in $X_{\tau }$ by simulating the null distribution of $X_{\tau }$ using gene dropping. We now derive the analytical mean and variance of *u *under the gene-dropping setting. In the score test using unrelated individuals, we treat *R *as random, and $X_{\tau }$ can be viewed as either random or fixed. In a gene-dropping simulation, *R *is held fixed, and $X_{\tau }$ is random.

Let i,j index individuals (i,j=1,…,n) and let $X_{\tau }={\left(X_{1},\ldots ,X_{n}\right)}^{\prime }$ and $R={\left(R_{1},\ldots ,R_{n}\right)}^{\prime }$. The expected value of *u *is $\overset{n}{\underset{i=1}{\sum }}E\left(X_{i}\right)R_{i}$ and $X_{i}=P_{i}+M_{i}$, where $P_{i}$$\left(M_{i}\right)$ is 1 if the paternal (maternal) allele is the minor allele and 0 otherwise. So $E\left(X_{i}\right)$ is twice the MAF $f_{\tau }$ at SNP *τ *and is the same for all individuals and thus $E\left(u\right)=2f_{\tau }\sum R_{i}=0$ because $R_{i}$'s are residuals from a linear regression model with intercept. The variance of *u *is $E\left(u^{2}\right)=R^{\prime }E\text{(}X_{\tau }X_{\tau }^{\prime })R.$ The (i,j) th element in $E\text{(}X_{\tau }X_{\tau }^{\prime })$ is $E\left(X_{i}X_{j}\right)=E\left(P_{i}P_{j}+P_{i}M_{j}+M_{i}P_{j}+M_{i}M_{j}\right).$$P_{i},M_{i},P_{j},M_{j}$ are all Bernoulli random variables with probability $f_{\tau }$, and any two of them are identical if the corresponding alleles are identity-by-descent (IBD) and are independent otherwise [8]. Let $\phi_{ij}$ be the number of IBD pairs among the four pairs of alleles $P_{i}P_{j},P_{i}M_{j},M_{i}P_{j},M_{i}M_{j}$. The value of $\phi_{ij}$ at locus *τ *is determined by the inheritance vector $S_{\tau }$, which summarizes whether the paternal or the maternal allele is passed from the parent to the child in each meiosis [9]. Given the inheritance vector $S_{\tau }$,

$$E\left(X_{i}X_{j}|S_{\tau }\right)=\phi_{ij}\left(S_{\tau }\right)f_{\tau }+\left(4-\phi_{ij}\left(S_{\tau }\right)\right)f_{\tau }^{2}=\phi_{ij}\left(S_{\tau }\right)\left(f_{\tau }-f_{\tau }^{2}\right)+4f_{\tau }^{2},$$

(e.g., $E\left(P_{i}P_{j}\right)=E\left(P_{i}^{2}\right)=f_{\tau }$ if $P_{i}$ and $P_{j}$ correspond to IBD alleles and $E\left(P_{i}P_{j}\right)=E\left(P_{i}\right)E\left(P_{j}\right)=f_{\tau }^{2}$ if $P_{i}$ and $P_{j}$ correspond to non-IBD alleles). In a gene-dropping simulation, the inheritance vector $S_{\tau }$ is randomly sampled among all possible inheritance vectors. The expected number of IBD alleles shared between *i *and *j*, $\text{E}\left(\phi_{ij}\left(S_{\tau }\right)\right),$ over all possible inheritance vectors is four times the kinship coefficient $\psi_{ij}\text{:E}\left(\phi_{ij}\left(S_{\tau }\right)\right)=4\psi_{ij}$. The kinship coefficients are determined by pedigree structures. The expected value of $X_{i}X_{j}$ in a gene-dropping simulation is thus $E\left(E\left(X_{i}X_{j}|S_{\tau }\right)\right)=4\psi_{ij}\left(f_{\tau }-f_{\tau }^{2}\right)+4f_{\tau }^{2}.$ Letting $\Phi \left(S_{\tau }\right)=\left(\phi_{ij}\right)$ be the matrix of IBD counts and *Ψ *be the matrix of kinship coefficients, we can rewrite the above as:

$$E\left(X_{\tau }{X^{\prime }}_{\tau }|S_{\tau }\right)=\Phi \left(S_{\tau }\right)\left(f_{\tau }-f_{\tau }^{2}\right)+4Jf_{\tau }^{2},$$

$$E\left(X_{\tau }X_{\tau }^{\prime }\right)=E\left(E\left(X_{\tau }X_{\tau }^{\prime }|S_{\tau }\right)\right)=4\Psi \left(f_{\tau }-f_{\tau }^{2}\right)+4Jf_{\tau }^{2},$$

where *J *is a matrix of all ones. Because $R^{\prime }JR=0$ for residuals from a linear regression model with an intercept, the variance of *u *under gene dropping is $v_{gd}=R^{\prime }E\left(X_{\tau }X_{\tau }^{\prime }\right)R=4R^{\prime }\Psi R\left(f_{\tau }-{f_{\tau }}^{2}\right)$ if unconditional on the inheritance vector $S_{\tau }$, and is $v_{\tau }=R^{\prime }E\left(X_{\tau }X_{\tau }^{\prime }|S_{\tau }\right)R=R^{\prime }\Phi \left(S_{\tau }\right)R\left(f_{\tau }-{f_{\tau }}^{2}\right)$ if conditional on the inheritance vector $S_{\tau }$ (holding $S_{\tau }$ fixed). We can approximate the gene-dropping null distribution of *u *by a normal distribution with mean 0 and variance $v_{gd}$, and compute the gene-dropping *p*-value by comparing $t=u^{2}/v_{gd}$ with a $\chi_{1}^{2}$ distribution. To test association in the presence of linkage, one needs to condition on the inheritance $S_{\tau }$ vector at *τ*[3] and use $v_{\tau }$. In practice, $S_{\tau }$ is not observable, but we estimate $v_{\tau }$ by drawing Markov chain Monte Carlo (MCMC) samples of $S_{\tau }$ based on observed genotypes in the pedigrees using MORGAN (http://www.stat.washington.edu/thompson/Genepi/MORGAN/Morgan.shtml) [10].

## Results

### Theoretical findings

In a gene-dropping simulation, the analytical mean of the score statistic $u={X_{\tau }}^{\prime }R$ is 0. The variance of the score statistic is $R^{\prime }\Phi \left({\text{S}}_{\tau }\right)R\left(f_{\tau }-f_{\tau }^{2}\right)$ if conditional on the inheritance vector (i.e., holding the inheritance vector fixed during gene-dropping simulation) and is $4R^{\prime }\Psi R\left(f_{\tau }-f_{\tau }^{2}\right)$ if unconditional on the inheritance vector. The normal approximation is justified by the central limit theorem because the test statistic is additive over pedigrees. Its performance depends on the number, sizes, and structure of pedigrees and on MAF at the test locus. The approximation may not be accurate for extremely small *p*-values. However, the rankings of the *p*-values will not change.

We can decompose the unconditional gene-dropping test statistic into two components:

$$\frac{u^{2}}{4R^{\prime }\Psi R\left(f_{\tau }-f_{\tau }^{2}\right)}=\left[\frac{u^{2}}{R^{\prime }\Phi \left({\text{S}}_{\tau }\right)R\left(f_{\tau }-f_{\tau }^{2}\right)}\right]\left[\frac{R^{\prime }\Phi \left({\text{S}}_{\tau }\right)R}{4R^{\prime }\Psi \text{R}}\right].$$

The first component can be used as a test statistic for detecting association in the presence of linkage (i.e., fine mapping under a linkage peak) because the denominator is the variance of *u *conditional upon the observed IBD sharing. The second component provides information about linkage. The kinship coefficients in *Ψ *are determined by pedigree structure, so $R^{\prime }\Psi R$ is a constant in a gene-dropping simulation. $R^{\prime }\Phi \left(S_{\tau }\right)R=\underset{ij}{\sum }r_{i}r_{j}\phi_{ij}\left(S_{\tau }\right)$ measures the correlation between trait value similarity $\left(r_{i}r_{j}\right)$ and IBD sharing $\left(\phi_{ij}\right)$ at locus *τ *across all pairs of individuals in a pedigree. This correlation is expected to be stronger if there is stronger linkage between *τ *and a true causal locus. Therefore, $R^{\prime }\Phi \left(S_{\tau }\right)R$ can be used as a test statistic to detect linkage, with null distribution obtained by gene-dropping simulations. In a gene-dropping simulation, the inheritance vectors are simulated as if they were from a marker unlinked to any potential causal loci. $R^{\prime }\Phi \left(S_{\tau }\right)R$ resembles similar quantities that are frequently used in linkage analysis methods such as the well-known Haseman-Elston regression [11] as well as many variance components or generalized estimating equation-based methods [12].

### Simulation results

We performed a genome-wide association studies (GWAS) score test using 142 unrelated individuals, the family-based association test using FBAT [3], and the gene-dropping test on SNPs on chromosome 3 (FBAT and the gene-dropping test used 847 individuals from 20 pedigrees). Table 1 summarizes the *p*-value ranks that each test assigns the true causal SNPs. The gene-dropping test for fine mapping (conditional on the inheritance vector) performs very similarly to the unconditional gene-dropping test, so its results are omitted. It is seen that the gene-dropping tests can quickly identify a few true causal SNPs within a short list of top findings. However, if we allow more false positives by considering a greater number of the most significant SNPs, other methods start to pick up true causal SNPs and eventually have a result similar to gene dropping.

**Table 1:** Ranks of truly influential single-nucleotide polymorphisms by genome-wide association studies, FBAT, and gene dropping

GWAS			FBAT			Gene dropping		
**Rank**	**Relative rank (%)**	**SNP position**	**Rank**	**Relative rank (%)**	**SNP position**	**Rank**	**Relative rank (%)**	**SNP position**

22	0.00212	47957996	1433	0.25642	47956424	1.5	0.00012	48040283
27	0.00260	48040283	2,903	0.51947	47958037	3.5	0.00029	47957996
1,561	0.15024	141693906	2,913	0.52126	50185967	202.5	0.01686	47958037
5,901	0.56796	47467805	5,536	0.99062	48040283	232	0.01932	47956424
11,415	1.09868	58161774	9,086.5	1.62595	47957996	3,937	0.32787	48040284
21,148.5	2.03552	47958037	15,860.5	2.83810	141093285	13,668	1.13826	58109162
23,791	2.28985	196597635	17,341.5	3.10311	141162128	19,870.5	1.65480	123170592
28,783	2.77033	135789360	22,148.5	3.96328	139276557	37,071	3.08725	141162128
30,761.5	2.96075	47956424	23,778	4.25487	141160882	40,497.5	3.37261	47913455
34,720.5	3.34180	58190853	32,483	5.81256	58192585	42,740	3.55936	141160882

Rank is raw ranks in terms of *p*-value significance of truly influential single-nucleotide polymorphisms (SNPs) (smaller numbers better, indicating that the method identifies a true SNP as more significant). The fractional ranks appearing in the gene-dropping column arise from ties: two SNPs being assigned exactly the same *p*-value. Note that it is not completely fair to compare these numbers directly because FBAT and genome-wide association studies (GWAS) produce not available (NA) results for a significant portion of the tested SNPs. Relative rank is the normalized ranks of truly influential SNPs: *p*-value rank divided by the total number of non-NA SNPs tested multiplied by 100. SNP position is the base-pair position of the identified truly influential SNP.

**Figure 1:**
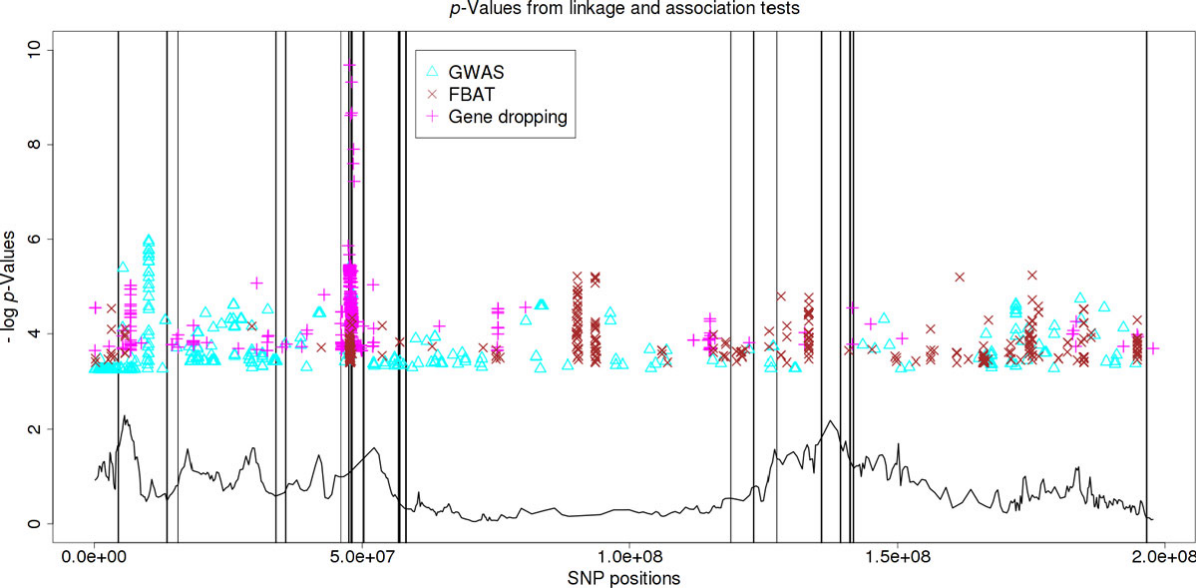
**p-Values from linkage and association tests. **Here we present the chromosome locations of the 500 most significant single-nucleotide polymorphisms (SNPs) on chromosome 3 identified by each method and their corresponding -log p-values. The triangles are the 500 most significant SNPs identified by the genome-wide association studies (GWAS) score test using unrelated individuals; the crosses are those identified by FBAT, and the plusses are those identified by the gene-dropping test. The solid curve shows the -log p-values from the linkage test at 449 evenly spaced SNPs (by comparing the linkage component of the gene-dropping test statistic with its Monte Carlo null distribution from gene dropping). Solid vertical lines indicate the positions of truly influential SNPs on chromosome 3.

Figure 1 shows the physical positions and negative log *p*-values of the top 500 SNPs identified by each of the three tests, as well as the negative log *p*-values of the linkage test based on the linkage component of the gene-dropping test statistic.

We also examined adjusting for population stratification by fitting the first two principal components of genetic variation [13] as covariates in the regression model (1). The *p*-values resulting from this expanded model differed negligibly from the original model. The ranks in Table 1 were essentially unchanged by this adjustment.

## Discussion

### Comparison between genome-wide association studies, FBAT and gene-dropping test

FBAT splits each pedigree into nuclear families. In each nuclear family, FBAT uses information from the offspring while conditioning on the parental marker genotypes. In contrast, GWAS uses information in unrelated individuals. The two methods use almost "orthogonal" sources of information. There is almost no correlation between the log *p*-values from these two methods (Table 2). In contrast, the gene-dropping test applies to multigeneration pedigrees and uses information from all individuals: the gene-dropping test extracts information from founders by resimulating founder genotypes and from offspring by resimulating inheritance vectors.

**Table 2:** Correlation between log *p*-values of genome-wide association studies, FBAT, and gene dropping

GWAS/FBAT	Gene dropping/FBAT	Gene dropping/GWAS
0.011	0.232	0.254

GWAS, genome-wide association studies.

It is also possible to derive the analytical mean and variance of the test statistic in the gene-dropping test where we permute the founder alleles rather than resimulate the founder alleles. FBAT is more robust to population stratification by conditioning on founder genotypes. The gene-dropping test can gain similar robustness by restricting permutations to founder alleles within each family.

It is somewhat surprising that the gene-dropping test did not outperform GWAS given that it uses more individuals. One possible interpretation is that the effect of LD is stronger when more individuals are used. As we can see in Figure 1, the signals detected by the gene-dropping test come in bigger clusters. In other words, many SNPs ranked high by the gene-dropping test might be in LD with one or more of the causal SNPs.

### Separating linkage and association signals

The gene-dropping test captures both linkage and association signals. One can decompose the test statistic into a linkage component and an association component.

The association component corresponds to testing association in the presence of linkage, which requires one to condition on the true inheritance vector at the test locus. Our results through MCMC approximation show that whether or not to condition on the inheritance vector actually does not make a big difference for this data set because the variance of the test statistic with conditioning only differs slightly from the variance of the test statistic without conditioning. This conclusion might be dependent on the structure of the pedigree.

The linkage component, however, clearly provides valuable information. The linkage signal is stronger in most regions containing causal SNPs. It is obvious that the linkage curve can help eliminate many of the false association signals in this study. It would be interesting to investigate how to use the linkage information more effectively in the future.

## Competing interests

The authors declare that they have no competing interests.

## Authors' contributions

All authors participated in analysis of the data. YJ, SE, and YD conceived of the study and drafted the manuscript. All authors participated in the critical revision of the manuscript and gave final approval of the article.

## References

[B1] MacCluer JW Vandeburg JL Read B Ryder OA Pedigree analysis by computer simulation Zoo Biol 1986 5 149 160

[B2] Allen-Brady K Wong J Camp NJ PedGenie: an analysis approach for genetic association testing in extended pedigrees and genealogies of arbitrary size BMC Bioinformatics 2006 7 209 10.1186/1471-2105-7-209 16620382 PMC1459209

[B3] Rabinowitz D Laird N A unified approach to adjusting association tests for population admixture with arbitrary pedigree structure and arbitrary missing marker information Hum Hered 2000 50 211 223 10.1159/000022918 10782012

[B4] Almasy L Dyer T Peralta J Jun G Fuchsberger C Almeida M Kent JW Jr Fowler S Duggirala R Blangero J Data for Genetic Analysis Workshop 18: human whole genome sequence, blood pressure, and simulated phenotypes in extended pedigrees BMC Proc 2014 8 suppl 2 S2 10.1186/1753-6561-8-S1-S2 PMC4145406 25519314

[B5] Di Y Mi G Sun L Dong R Zhu H Peng L Power of association tests in the presence of multiple causal variants BMC Proc 2011 5 suppl 9 S63 10.1186/1753-6561-5-S9-S63 22373395 PMC3287902

[B6] Schaid DJ Rowland CM Tines DE Jacobson RM Poland GA Score tests for association between traits and haplotypes when linkage phase is ambiguous Am J Hum Genet 2002 70 425 434 10.1086/338688 11791212 PMC384917

[B7] Clayton D Chapman J Cooper J Use of unphased multilocus genotype data in indirect association studies Genet Epidemiol 2004 27 415 428 10.1002/gepi.20032 15481099

[B8] Lange K Westlake J Spence MA Extensions to pedigree analysis. III. Variance components by the scoring method Ann Hum Genet 1976 39 485 491 10.1111/j.1469-1809.1976.tb00156.x 952492

[B9] Lander ES Green P Construction of multilocus genetic linkage maps in humans Proc Natl Acad Sci USA 1987 84 2363 2367 10.1073/pnas.84.8.2363 3470801 PMC304651

[B10] Thompson EA Liang F, Wang J-S, Kendall W MCMC in the analysis of genetic data on pedigrees Markov Chain Monte Carlo: Innovations and Applications Lecture Note Series of the IMS, National University of Singapore. World Scientific Co Pte Ltd, Singapore 183 216

[B11] Elston RC Buxbaum S Jacobs KB Olson JM Haseman and Elston revisited Genet Epidemiol 2000 19 1 17 10.1002/1098-2272(200007)19:1<1::AID-GEPI1>3.0.CO;2-E 10861893

[B12] Chen WM Broman KW Liang KY Quantitative trait linkage analysis by generalized estimating equations: unification of variance components and Haseman-Elston regression Genet Epidemiol 2004 26 265 272 10.1002/gepi.10315 15095386

[B13] Price AL Patterson NJ Plenge RM Weinblatt ME Shadick NA Reich D Principal components analysis corrects for stratification in genome-wide association studies Nat Genet 2006 38 904 909 10.1038/ng1847 16862161

